# Rice Hypersensitive Induced Reaction Protein 1 (OsHIR1) associates with plasma membrane and triggers hypersensitive cell death

**DOI:** 10.1186/1471-2229-10-290

**Published:** 2010-12-30

**Authors:** Liang Zhou, Ming-Yan Cheung, Man-Wah Li, Yaping Fu, Zongxiu Sun, Sai-Ming Sun, Hon-Ming Lam

**Affiliations:** 1 State Key Laboratory of Agrobiotechnology and School of Life Sciences, The Chinese University of Hong Kong, Shatin, Hong Kong SAR, PR China; 2 State key Laboratory of Rice Biology, China National Rice Research Institute, Hangzhou, Zhejiang, PR China

## Abstract

**Background:**

In plants, HIR (Hypersensitive Induced Reaction) proteins, members of the PID (Proliferation, Ion and Death) superfamily, have been shown to play a part in the development of spontaneous hypersensitive response lesions in leaves, in reaction to pathogen attacks. The levels of HIR proteins were shown to correlate with localized host cell deaths and defense responses in maize and barley. However, not much was known about the HIR proteins in rice. Since rice is an important cereal crop consumed by more than 50% of the populations in Asia and Africa, it is crucial to understand the mechanisms of disease responses in this plant. We previously identified the rice HIR1 (OsHIR1) as an interacting partner of the OsLRR1 (rice Leucine-Rich Repeat protein 1). Here we show that OsHIR1 triggers hypersensitive cell death and its localization to the plasma membrane is enhanced by OsLRR1.

**Result:**

Through electron microscopy studies using wild type rice plants, OsHIR1 was found to mainly localize to the plasma membrane, with a minor portion localized to the tonoplast. Moreover, the plasma membrane localization of OsHIR1 was enhanced in transgenic rice plants overexpressing its interacting protein partner, OsLRR1. Co-localization of OsHIR1 and OsLRR1 to the plasma membrane was confirmed by double-labeling electron microscopy. Pathogen inoculation studies using transgenic *Arabidopsis thaliana *expressing either OsHIR1 or OsLRR1 showed that both transgenic lines exhibited increased resistance toward the bacterial pathogen *Pseudomonas syringae *pv. *tomato *DC3000. However, *OsHIR1 *transgenic plants produced more extensive spontaneous hypersensitive response lesions and contained lower titers of the invading pathogen, when compared to *OsLRR1 *transgenic plants.

**Conclusion:**

The OsHIR1 protein is mainly localized to the plasma membrane, and its subcellular localization in that compartment is enhanced by OsLRR1. The expression of OsHIR1 may sensitize the plant so that it is more prone to HR and hence can react more promptly to limit the invading pathogens' spread from the infection sites.

## Background

In plants, there are no immune cells against invading pathogens. Nonetheless, they have evolved different strategies for defense [1,2]. The current model depicts that plants can recognize pathogen-associated molecular patterns (PAMPs) to trigger an immune response. If such a defense mechanism is compromised by effectors produced by the pathogens, host plants that possess resistance proteins which can recognize the effectors will still be able to trigger an immune response. Both PAMP-triggered and effector-triggered immunities may result in hypersensitive response (HR), which is characterized by the rapid and localized responses that lead to the generation of reactive oxygen species, cell wall fortification and a special form of programmed cell death (PCD), also known as hypersensitive cell death [3-5]. PCD is an important mechanism of removing unwanted cells in order to model or remodel newly-forming organs [6-8]. Stress-induced PCD in both plant and animal cells may involve the endomembrane system [9].

HR involves the expression of genes and the *de novo *synthesis of proteins that are part of several defense response signaling pathways [4,10,11]. HR-like lesions can be induced in the absence of pathogens by overexpressing defense-related genes [4,12-14]. These genes can be categorized into 4 classes: pathogen-derived genes, genes involved in defense signal transduction, killer genes, and general metabolism-perturbing genes [13]. Furthermore, plants exhibiting transgene-induced cell death are also resistant to pathogen infection by activating the defense signaling pathways [11,13].

Hypersensitive Induced Reaction (HIR) proteins are a group of proteins involved in HR. They belong to the PID (Proliferation, Ion and Death) superfamily, whose members function in cell proliferation, ion channel regulation and cell death [15]. HIR protein expression in maize and barley is associated with localized host cell death and disease resistance responses [15,16]. Their genes are up-regulated in plant leaves during the development of spontaneous HR lesions [15-17].

Rice is an important cereal that provides calories to more than 50% of the Asian and African populations. However, rice production has suffered from various pathogenic attacks [1]. While HIR proteins from other cereals have been shown to be involved in defense responses [15,16], the information on the HIR proteins in rice is very limited. We previously identified the rice HIR1 (OsHIR1) as the interacting partner of the rice Leucine-Rich Repeat protein 1 (OsLRR1) via yeast two-hybrid and *in vitro *pull-down experiments [18]. OsLRR1 enters the endosomal pathway and its ectopic expression in transgenic *Arabidopsis thaliana *can enhance the host resistance toward the virulent pathogen *Pseudomonas syringae *pv. *tomato *(*Pst*) DC3000 [18].

In this study, we provide evidence to show that OsLRR1 enhances the plasma membrane localization of OsHIR1. We also demonstrate the involvement of OsHIR1 in triggering hypersensitive cell death and plant defense response using transgenic *A. thaliana*.

## Results

### *OsHIR1 *encodes a Band 7-domain protein which is up-regulated upon pathogen challenge

**Figure 1:**
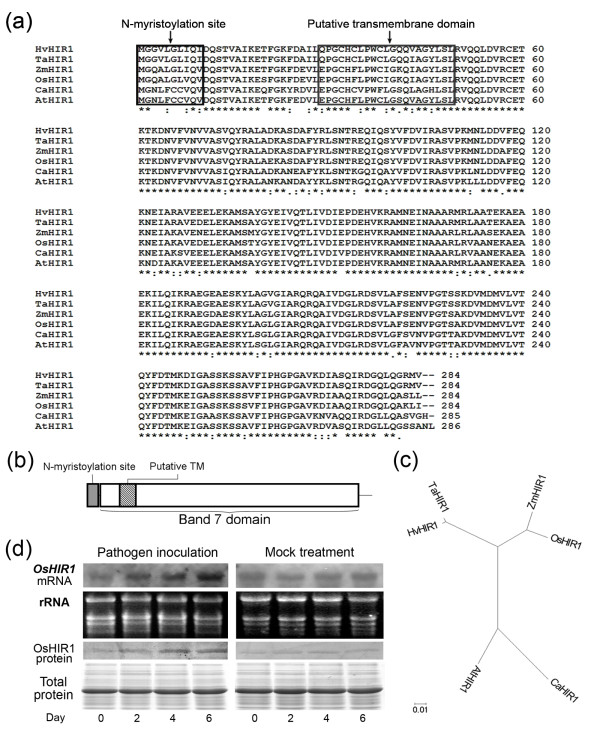
**Structural domains and phylogenetic relationships of OsHIR1 homologues and expression of OsHIR1 under pathogen inoculation**. (a) Alignment of OsHIR1 homologues in plants. "*" represents conserved amino acid residues, ":" conserved substitutions, and "." semi-conserved amino acid substitutions. (b) Schematic representation of the conserved structural domains in OsHIR1 and its homologues. (c) Phylogenetic analysis of OsHIR1 and its published plant homologues. (d) The mRNA and protein levels of OsHIR1 0, 2, 4 and 6 days after inoculation of *Xanthomonas oryzae *pv. *oryzae *(*Xoo*) race LN44 or mock treatment by a leaf-clipping method. Ten μg total RNA and 10 μg total protein were loaded onto each lane.

OsHIR1 was identified as a putative interacting partner of OsLRR1 [18]. The OsHIR1 protein exhibits high similarity (from 84% to 96% identity) to homologues from dicots and monocots (Figure 1a), including maize (*Zea mays*) [15], barley (*Hordeum vulgare *subsp. *Vulgare*) [16], wheat (*Triticum aestivum*) [19], pepper (*Capsicum *spp.) [20], and *A. thaliana *[21,22]. For all the close homologues of OsHIR1, computational analysis [23,24] reveals a putative N-myristoylation site at the N-terminus, followed by a transmembrane domain that is embedded within a Band 7-domain, which covers most of the OsHIR1 protein (Figure 1b). In an unrooted phylogenetic tree (Figure 1c), HIR proteins can be further divided into two branches: dicots and monocots. Among HIR homologues from monocots, the OsHIR1 shares the highest similarity with the maize ZmHIR1 (96% identity).

To show that OsHIR1 is related to the plant defense response, we investigated whether its gene expression is responsive to pathogen challenge. Northern and western blot analyses showed that both the mRNA and protein levels of OsHIR1 increased after the rice plant was inoculated with the pathogen *Xoo *LN44 (Figure 1d). On the other hand, no such change was observed after mock treatment (Figure 1d).

### Subcellular localization of OsHIR1 and the possible interaction with OsLRR1

**Figure 2:**
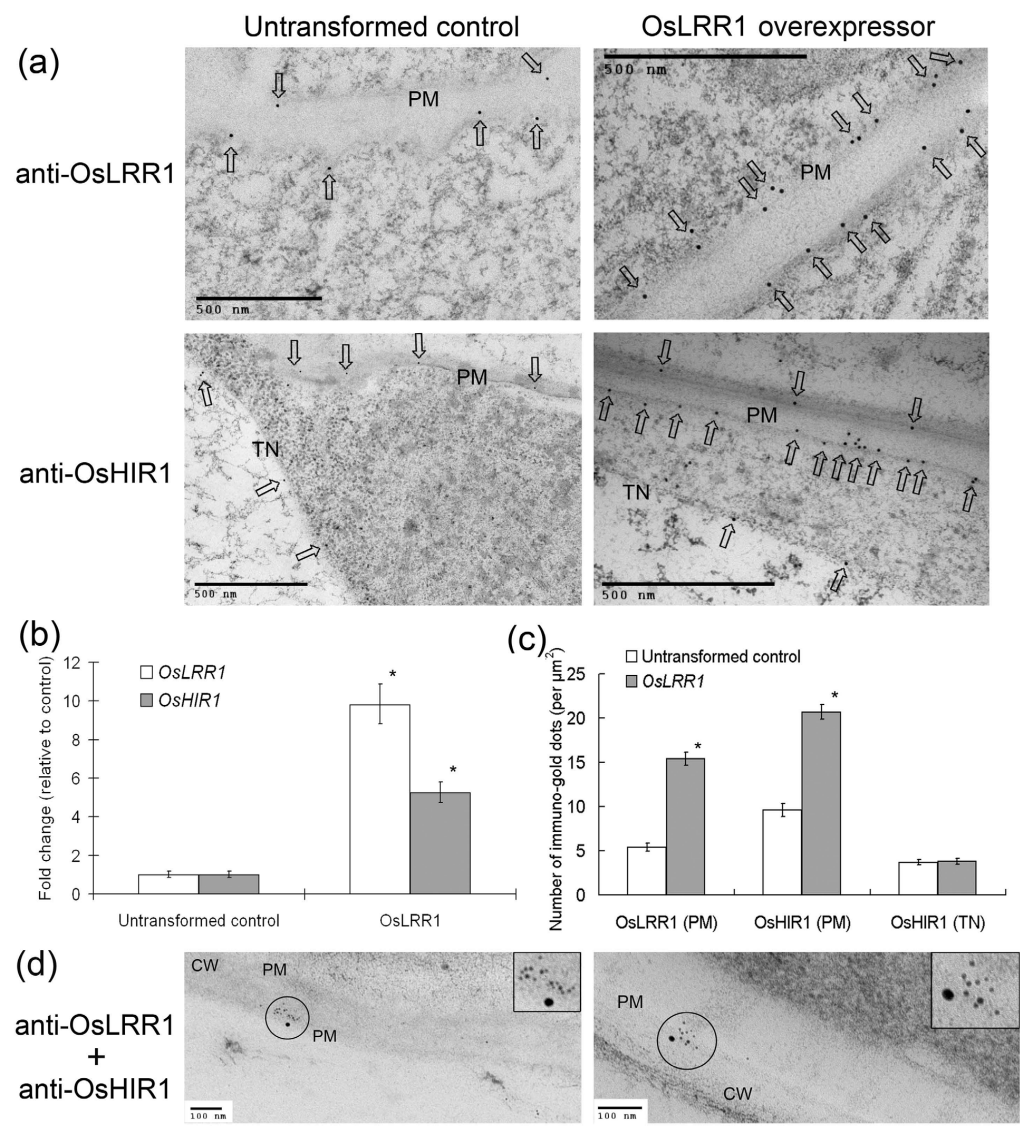
**Regulation of the subcellular localization of OsHIR1 by OsLRR1**. (a) Immuno-gold electron microscopy studies. Anti-OsLRR1 and anti-HIR1 antibodies were used to detect the subcellular localization of OsLRR1 and OsHIR1, respectively, in rice leaves. PM: Plasma membrane; TN: Tonoplast. (b) Expression of *OsLRR1 *and *OsHIR1 *in an *OsLRR1 *overexpressing rice line. Real-time RT-PCR analysis was performed to compare the relative gene expression (expression in untransformed control was set to 1). Error bars show the standard errors (N = 3). (c) Semi-quantitative analysis of OsHIR1 and OsLRR1 electron microscopy signals in the untransformed control and the *OsLRR1 *overexpressing rice line. The immuno-gold-labeled signal counting was described in Methods. Error bars show the standard errors (N = 10). * in (b) and (c) indicates that the difference is significant (*p *< 0.05, Student's *t*-test) between the transformants and the untransformed wild type. (d) Double labeling of OsHIR1 and OsLRR1. Two independent photos were shown to illustrate the co-localization of OsHIR1 (15 nm gold particles) and OsLRR1 (6 nm gold particles) to the plasma membrane. PM: Plasma membrane; CW: Cell wall.

We previously reported that the OsHIR1 proteins were retained in the membrane-associated protein fraction and might be localized to the plasma membrane [18]. However, a more detailed electron microscopy analysis showed that a minor portion of OsHIR1 signals could also be found to the tonoplast (Figure 2a, lower left panel).

To study the possible effects of OsLRR1 on the subcellular localization of OsHIR1, we constructed transgenic rice lines overexpressing OsLRR1. A transgenic line that exhibited a high level of *OsLRR1 *gene expression was chosen for subsequent electron microscopy analysis (Figure 2b). Interestingly, in addition to the elevated level of *OsLRR1 *mRNA, the expression of the *OsHIR1 *gene in the *OsLRR1 *transgenic line was also enhanced (Figure 2b).

Immuno-gold electron microscopy studies showed that not only the signal density of the OsLRR1 proteins, but also that of the OsHIR1 proteins, in the plasma membrane, was increased in the *OsLRR1 *overexpressing line by at least two folds, when compared to the untransformed control (Figure 2c). On the other hand, there was no significant difference (Student's t-test, *p *< 0.05) between the number of OsHIR1 signals in the tonoplast of the *OsLRR1 *overexpressing line and that in the untransformed control. These results indicated that OsLRR1 enhanced the plasma membrane localization of OsHIR1.

To further confirm the *in vivo *interaction between OsHIR1 and OsLRR1 in the plasma membrane, a double labeling experiment was performed using rabbit anti-OsLRR1 antibodies and mouse anti-OsHIR1 antibodies. Secondary antibodies conjugated with gold particles of different sizes (6 nm anti-rabbit IgG and 15 nm anti-mouse IgG) were employed to distinguish between the two target proteins. Proximal occurrences of large and small gold particles were detected in the plasma membrane (Figure 2d), supporting the notion that OsLRR1 and OsHIR1 co-localized and interacted in the plasma membrane.

### Ectopic expression of the OsHIR1 can cause spontaneous hypersensitive response lesions in the leaves of transgenic *A. thaliana*

**Figure 3:**
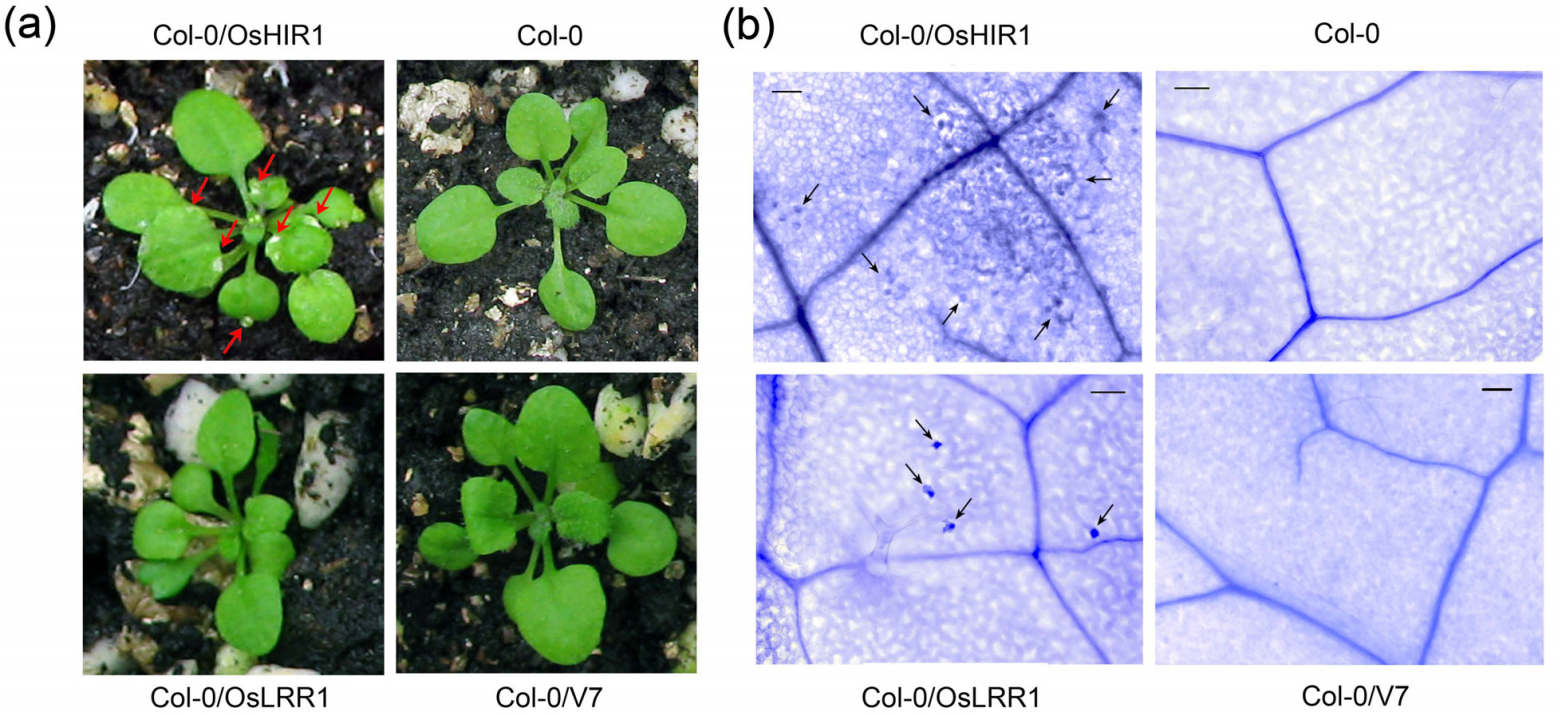
**Hypersensitive response lesions and spontaneous cell death due to the overexpression of OsHIR1**. (a) Hypersensitive response lesions in some *OsHIR1 *transgenic plants. Three weeks after germination, white necrotic lesions located randomly at the margins and tips of leaves (red arrows) were observed in about 20% of the *OsHIR1 *transgenic plants. Such a phenomenon was not found in untransformed wild type (Col-0), empty vector transgenic control (Col-0/V7), or *OsLRR1 *transgenic plants (Col-0/OsLRR1). (b) Lactophenol-trypan blue staining showing spontaneous cell death. Leaves of 3-week-old plants were stained with lactophenol-trypan blue to detect dead cells. Spontaneous cell death found on the leaves of *OsHIR1 *and *OsLRR1 *transgenic plants were indicated by black arrows. Bars = 100 μm

To perform a rapid gain-of-function test of OsHIR1, transgenic *A. thaliana *plants ectopically expressing OsHIR1 were generated. Three weeks after germination, the leaves of about 20% of the *OsHIR1 *transgenic plants (Col-0/OsHIR1) exhibited white spontaneous HR lesions located randomly at the margins and tips (Figure 3a, red arrows). As negative controls, the untransformed wild type (Col-0) and transgenic plants with the empty vector (Col-0/V7) exhibited normal growth. Transgenic plants expressing OsLRR1 (Col-0/OsLRR1) did not exhibit visible differences in the size, shape, or color of the leaves, when compared to the negative controls (Figure 3a).

To further observe the effect of OsHIR1 on cell death, lactophenol-trypan blue staining was performed using the leaves of the transgenic *A. thaliana*. The expression of OsHIR1 caused extensive spontaneous cell death (Figure 3b, black arrows). On the other hand, the expression of OsLRR1 only resulted in very mild spontaneous cell death (Figure 3b). This explains the lack of visible lesions found in *OsLRR1 *transgenic plants (Figure 3a). No spontaneous cell death was observed in the untransformed control and transgenic plants containing the empty vector (Figure 3b).

### Ectopic expression of OsHIR1 in transgenic *A. thaliana *enhances resistance to *P. syringae *pv. *tomato *DC3000 (*Pst *DC3000)

Previous studies indicated that the ectopic expression of OsLRR1, the interacting protein partner of OsHIR1, can enhance resistance toward bacterial pathogens in transgenic *A. thaliana *[18]. Using a similar experimental approach, we tested the effects of OsHIR1 in *A. thaliana *on the *Pst *DC3000-induced disease. Since *OsHIR1 *transgenic plants exhibiting extensive spontaneous HR responses under normal growth conditions would eventually die, we chose those individual plants that exhibited the mildest spontaneous HR responses for the subsequent pathogen inoculation tests. The expression of *OsHIR1 *in these plants was confirmed by RT-PCR (data not shown).

**Figure 4:**
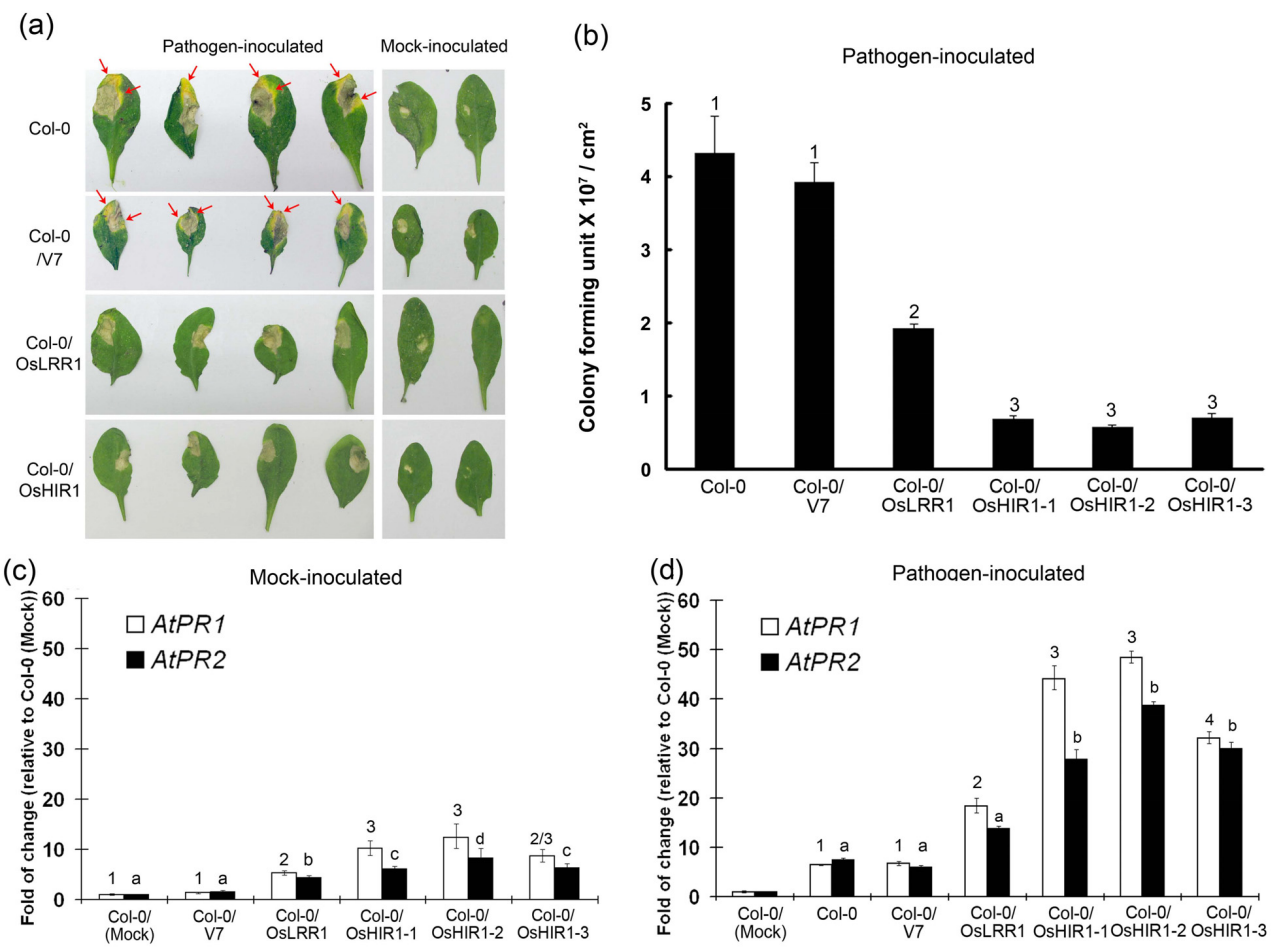
**Pathogen inoculation test of transgenic *A. thaliana *expressing OsHIR1**. (a) Disease symptoms after pathogen inoculation. Six-week-old seedlings of the untransformed wild type (Col-0), the empty vector-transformed control (Col-0/V7), and the *OsLRR1 *(Col-0/OsLRR1) and *OsHIR1 *transgenic lines (Col-0/OsHIR1) were challenged with *Pst *DC3000. The symptoms were recorded 5 days after inoculation. (b) Pathogen titers 5 days after pathogen inoculation. Rosette leaves were collected from inoculated plants for pathogen titer determination. Statistical analysis using ANOVA followed by Fisher's LSD Test (*p *< 0.05) reveals 3 groups: ^1^, the untransformed wild type and the vector-only control; ^2^, *OsLRR1 *transgenic plants; and ^3^, *OsHIR1 *transgenic plants. The error bars indicate standard errors (N = 3). (c) and (d) Expression of defense marker genes without (mock) or with *Pst *DC3000 inoculation. Real-time RT-PCR was performed using reverse-transcribed RNA samples. Relative expression levels of *PR1 *and *PR2 *in all plants were compared to the mock-inoculated untransformed wild type parent (Col-0; expression level set to 1). Both the expressions of *PR1 *and *PR2 *can be categorized into different groups using ANOVA followed by Fisher's LSD Test (*p *< 0.05). In (d), the gene expression in mock-treated Col-0 was used just to set the reference for gene expression and was not included in the statistical analysis. The error bars indicate standard errors (N = 3). Three independent *OsHIR1 *transgenic lines (Col-0/HIR1-1, Col-0/HIR1-2, and Col-0/HIR1-3) were used for quantitative studies in (b), (c), and (d).

When the untransformed wild type (Col-0) or *A. thaliana *transformed with the empty vector cassette (Col-0/V7) was inoculated with the pathogen *Pst *DC3000, disease symptoms (yellowing and necrosis) gradually appeared and the infected areas spread out from the original inoculation sites (Figure 4a). Such symptoms were alleviated in the transgenic line expressing OsLRR1, consistent with the results of our previous study [18]. The spread of pathogen infection was also suppressed by the ectopic expression of OsHIR1 (Figure 4a). Consistent with these visible symptoms, transgenic plants expressing either OsLRR1 or OsHIR1 exhibited a lower titer of pathogens when compared to Col-0 and the empty vector control (Figure 4b). However, the *OsHIR1 *transgenic lines showed a stronger effect on lowering the pathogen titer when compared to the *OsLRR1 *transgenic line (Figure 4b).

The expression levels of *PR1 *and *PR2*, two defense marker genes in the salicyclic acid pathway related to the defense against biotrophic pathogens such as *Pst *DC3000 [25], were monitored in both mock- (Figure 4c) and pathogen-inoculated (Figure 4d) plants. In both mock-treated and pathogen-inoculated plants, the expression levels of *PR1 *and *PR2 *were elevated in both *OsHIR1 *and *OsLRR1 *transgenic plants when compared to Col-0 and transgenic plants containing the empty vector cassette. However, the *OsHIR1 *transgenic plants exhibited significantly higher levels of *PR1 *and *PR2 *gene induction than the *OsLRR1 *transgenic plants (*p *< 0.05).

## Discussion

OsHIR1 is a member of the Band 7-domain-containing proteins (Figure 1). Many of these proteins are lipid raft-associated and may cluster to form membrane micro-domains, and in turn recruit multi-protein complexes functioning in membrane trafficking and signal transduction [26]. Signaling components found in plasma membrane lipid rafts may play important roles in defense responses. For example, an E3 ubiquitin ligase, RING1, is induced by pathogen infection, localizes to plasma membrane lipid rafts, and can trigger programmed cell death in *A. thaliana *[27].

Here the membrane localization of OsHIR1 was confirmed with electron microscopy studies (Figure 2). We also showed that OsHIR1 and OsLRR1 co-localized to the plasma membrane (Figure 2), possibly via lipid rafts. This result further confirms the tight interaction between OsHIR1 and OsLRR1 previously shown by yeast two-hybrid and *in vitro *pull-down assays [18]. Overexpressing OsLRR1 can induce the expression of *OsHIR1 *gene and can increase the portion of OsHIR1 localized to the plasma membrane (Figure 2). Therefore, it is likely that the function of OsHIR1 is regulated by its interacting partner OsLRR1.

It is an interesting observation that a minor portion of OsHIR1 is localized to the tonoplast (Figure 2). Although it has not been explicitly discussed in previous researches, proteomics studies have identified rice and Arabidopsis HIR1 homologues in both the plasma membrane and vacuole protein fractions [21,22,28-31]. A recent report showed that the vacuolar contents discharged and accumulated in the extracellular space could induce hypersensitive cell death [32]. However, the biological significance of the tonoplast localization of OsHIR1 remains unclear at this point.

OsLRR1 is a positive signaling component of plant defense responses [18]. The regulatory actions of OsLRR1 on the expression and localization of OsHIR1 suggest that OsHIR1 may be downstream of OsLRR1 in a defense response pathway. Previous studies of HIR1 homologues from maize, barley, and pepper indicated that they are associated with HR and disease resistance [15,16,20].

In transgenic *A. thaliana *ectopically expressing OsHIR1, a portion of plants underwent uncontrolled spontaneous HR (Figure 3) and eventually died. *OsHIR1 *transgenic plants with the mildest spontaneous HR phenotype could survive and were more resistant to the bacterial pathogen *Pst *DC3000. The protective effects of OsHIR1 included the alleviation of disease symptoms, the lowering of pathogen titers, and the increased expression of defense marker genes. Similar effects could be obtained by expressing OsLRR1, the interacting protein partner of OsHIR1 [18] (Figure 4). In general, OsHIR1 showed a stronger enhancing effect on disease resistance when compared to OsLRR1. In the native system, OsLRR1, which is trafficked in the endosomal pathway, may participate in the surveillance of pathogen-related signals and then induce the production and regulate the plasma membrane localization of OsHIR1. It is likely that the protective function of OsLRR1 is at least in part mediated through OsHIR1.

## Conclusion

The OsHIR1 protein identified in rice is mainly localized to the plasma membrane where it may co-localize and interact with the OsLRR1 protein. The overexpression of OsLRR1 can enhance the plasma membrane localization of OsHIR1. Ectopic expression of either OsHIR1 or OsLRR1 can cause spontaneous hypersensitive cell death and increased resistance toward bacterial pathogens, with OsHIR1 demonstrating a more pronounced effect than OsLRR1. We speculate that the expression of OsHIR1 may sensitize the plant so that it is more prone to HR and hence can react more promptly to restrict the spread of the invading pathogens from the infection sites. OsLRR1 may act as a regulator for the functions of OsHIR1.

## Methods

### Plant materials, chemicals, reagents and primers

*A. thaliana *wild-type Col-0 and *Oryza sativa *cultivar SN1033 are laboratory stocks. The *Pseudomonas syringae *pv. *tomato *DC3000 (*Pst *DC3000) was a gift from Dr. C. Lo (HKU). Enzymes and reagents for molecular studies were from Applied Biosystems (Foster City, CA), Clontech Laboratories, Inc. (Palo Alto, CA), Bio-Rad Laboratories (Hercules, CA), Promega Biosciences (San Luis Obispo, CA), and Roche Diagnostic Ltd (Basel, Switzerland). DNA oligos were from Integrated DNA Technologies, Inc. (Coraliville, IA), Invitrogen Corp. (Carlsbad, CA), and Tech Dragon Ltd. (Hong Kong). Chemicals for plant growth and tissue cultures were from Sigma-Aldrich Co. (St Louis, MO). The soil for *A. thaliana *cultivation was from Florgard Vertriebs GmbH (Gerhard-Stalling, Germany).

### RNA extraction, cDNA preparation, real-time PCR and northern blot analysis

RNA extraction, cDNA preparation, and real-time PCR were performed as previously described [18,33-35]. For real-time PCR, at least two biological repeats were performed. All experiments were done with at least four technical replicates and at least three sets of consistent data were used for analysis. The expression levels of the *A. thaliana UBQ10 *gene (*AtUBQ10*; GenBank accession number AY139999; [36]) with the primer set 5'-GGCCTTGTATAATCCCTGATGAATAAG-3' and 5'-AAAGAGATAACAGGAACGGAAACATAGT-3' and the *O. sativa UBQ5 *gene (*OsUBQ5*; GenBank accession number AK061988; [37]) with the primer set 5'-ACCACTTCGACCGCCACTACT-3' and 5'-ACGCCTAAGCCTGCTGGTT-3' were used for normalization in *A. thaliana *and *O. sativa *respectively. The relative gene expression was calculated using the 2^-ΔΔCT ^method [38].

Other primer sets for real-time PCR studies include *AtPR1*: 5'-AACTACAACTACGCTGCGAACAC-3' and 5'-CTTCTCGTTCACATAATTCCCAC-3'; *AtPR2*: 5'-CGCCCAGTCCACTGTTGATA-3' and 5'-ACCACGATTTCCAACGATCC-3'; and *OsHIR1*: 5'-CCCTGGTGCATAGGGAAGCA-3' and 5'-CGTCTG ATGCCTTCTCAGCAA-3'.

Northern blot analyses were performed as described [33,35] using antisense single-stranded DNA probes labeled with digoxygenin (DIG) (Roche, Germany) [39].

### Plant growth and pathogen inoculation

Rice lines were grown on soil in a greenhouse under natural sunlight for 4 to 5 weeks. Pathogen inoculations were performed using *Xanthomonas oryzae *pv. *oryzae *(*Xoo*) race LN44 by a leaf-clipping method [34,40,41]. The same procedure was used for mock treatment except that the pathogen was replaced with water. The day 0 sample was collected before treatment. Other samples were collected at 2, 4, and 6 days after treatment at around the same time of day (between 08:00 and 10:00 am).

For pathogen inoculation tests in *A. thaliana*, seedlings were first grown on Murashige & Skoog salt mixture agar plates for 2 weeks before being transferred to Floragard potting soil and cultivated in a growth chamber (22-24°C; relative humidity 70-80%; light intensity 80-120 μE on a 16 h light-8 h dark cycle). Preparation of the *Pst *DC3000 culture, inoculation (by syringe infiltration of 0.1 ml inoculums at a concentration of 10^6 ^colony-forming unit/ml in 10 mM MgSO_4 _supplemented with 0.02% (v/v) Silwet L-77), and subsequent pathogen titer determination at 5 days post-inoculation were performed as previously described [42]. For the pathogen titer measurement, leaf discs were macerated and extracted with 10 mM MgSO_4_, and the results were obtained from plate counting [42]. Error bars are standard errors of the pathogen titer calculated from samples collected from 3 individual plants each consists of 3 leaf discs.

### Transgenic plant construction

To construct transgenic rice lines overexpressing OsLRR1, the full-length coding region of OsLRR1 was subcloned into the binary vector pSB130 [43], using the primer set 5'- CCGAATTCATGGGGGCGGGGGCGCTG-3' and 5'-CAGGTCGACGCTAGCAGTTGGTGTCATATACAG-3'. Constitutive expression was driven by the *Zea mays *ubiquitin promoter [44]. The recombinant construct was introduced into the japonica rice SN1033 via an *Agrobacterium*-mediated protocol [45,46] using the *A. tumefaciens *strain EHA105.

Transgenic *A. thaliana *expressing OsLRR1 was from our previous work [18]. To construct transgenic *A. thaliana *expressing OsHIR1, a cDNA clone containing the full-length coding region was inserted into a binary vector (V7; [47]) and placed under the control of the cauliflower mosaic virus 35S promoter using the primer set 5'-AGTTCTAGAATGGGTCAAGCACTCGGTTTGGTAC-3' and 5'-AAAAATCTA GATTAGATCAATTTGGCCTGGAGCTG-3'. *Agrobacterium*-mediated transformation of *A. thaliana *was done as described previously [48]. T3 homozygous lines carrying a single insertion locus were used in this study.

### Electron microscopy studies

For single labeling experiments, the embedding, sectioning, and immunolabeling steps were performed as described [18,49] using mouse anti-OsHIR1 serum or rabbit anti-OsLRR1 serum [18]. All the sections were captured by formvar-coated 100 mesh hex nickel grid (Cat. No. G100H-Ni, Electron Microscopy Sciences). The subcellular localization of targeted proteins were subsequently detected by gold-labeled secondary antibodies (1:50 in 1% PBS-BSA) against mouse (EMS25173) or rabbit (EMS25109) IgGs. Aqueous uranyl acetate/lead citrate post-stained sections were examined with the Hitachi H-7650 transmission electron microscope operating at 80 kV. Background signals were monitored by negative control experiments without the application of the primary antibodies [18]. All images were captured at regions showing clear plasma membrane and tonoplast, with the magnification between 50,000× to 80,000×. At least ten randomly selected areas (1-2 μm^2^) per section were used for counting the density of immuno-gold-labeled dots (number of dots per μm^2^) for statistical analysis.

For double labeling experiments, tissues were collected from the untransformed control. Sample preparation, labeling, post-staining, and detection procedures were the same as in single labeling experiments, except that rabbit anti-OsLRR1 serum and mouse anti-OsHIR1 serum (both 1:50 in 1% PBS-BSA) were applied simultaneously to the sample grid to detect the target proteins. Goat anti-rabbit IgG (6 nm gold particle: EMS 25104) and goat anti-mouse IgG + IgM (15 nm gold particle: EMS 25173) were applied simultaneously to detect the primary antibodies.

### Western blot analysis

Total proteins were extracted [49] and electrophoretically separated on an SDS-polyacrylamide gel (4% stacking; 12.5% resolving) before being transferred to an activated polyvinylidene difluoride (PVDF) membrane pre-treated with absolute methanol for 5 min followed by protein transfer buffer for another 5 min, using the Bio-Rad Mini Trans-Blot^® ^Electrophoretic Transfer Cell (170-3930; Bio-Rad). The blotting, blocking (with Western Breeze™ blocking solution), and detection (using the Western Breeze™ Immunodetection Kit; WB7106, Invitrogen) procedures were performed according to the manufacturer's manuals.

Primary antibodies against the OsHIR1 protein [18] were used. Anti-mouse secondary antibodies conjugated to an alkaline phosphatase (provided in the Western Breeze™ Immunodetection Kit) were used for primary antibody recognition.

### Lactophenol-trypan blue staining

Spontaneous cell death was detected using lactophenol-trypan blue staining as previously described [50].

### Bioinformatics analysis

Alignment of amino acid sequences was done using the ClustalW2 program http://www.ebi.ac.uk/Tools/clustalw2/. The GenBank accession numbers of HIR1 homologues in this work are: rice OsHIR1 (accession no. NM_001068279), barley HvHIR1 (accession no. AY137511), wheat TaHIR1 (accession no. EF514209), maize ZmHIR1 (accession no. NM_001112153), pepper CaHIR1 (accession no. AY529867), and Arabidopsis AtHIR1 (accession no. NM_125669). The putative N-myristoylation site was predicted by ScanProsite [23] and CSS-Palm 2.0 [24]. The putative transmembrane domain was predicted by TopPred [51].

### Statistical analysis

Statistical analyses were performed using Statistical Package for Social Sciences v. 15.0.

## Authors' contributions

ZL carried out most of the experimental works. MYC prepared the recombinant construct for making transgenic rice, rice RNA samples for gene expression studies, and performed EM studies with double labeling together with MWL. YF and ZS generated the transgenic rice lines. HML coordinated the design, data analysis, and execution of this study. SMS participated in the experimental design. HML, ZL, MYC, and MWL wrote the manuscript. All authors read and approved the final manuscript.

## References

[B1] Jones JDG Dangl JL The plant immune system Nature 2006 444 323 329 10.1038/nature05286 17108957

[B2] Pieterse CMJ Leon-Reyes A Van der Ent S Van Wees SCM Networking by small-molecule hormones in plant immunity Nat Chem Biol 2009 5 308 316 10.1038/nchembio.164 19377457

[B3] Greenberg JT Programmed cell death in plant-pathogen interactions Ann Rev Plant Physiol Plant Mol Biol 1997 48 525 545 10.1146/annurev.arplant.48.1.525 15012273

[B4] Greenberg JT Yao N The role and regulation of programmed cell death in plant-pathogen interactions Cell Microbiol 2004 6 201 211 10.1111/j.1462-5822.2004.00361.x 14764104

[B5] van Doorn WG Woltering EJ Many ways to exit? Cell death categories in plants Trends Plant Sci 2005 10 117 122 10.1016/j.tplants.2005.08.003 15749469

[B6] Giuliani C Consonni G Gavazzi G Colombo M Dolfini S Programmed cell death during embryogenesis in maize Ann Bot 2002 90 287 292 10.1093/aob/mcf173 12197527 PMC4240416

[B7] Gunawardena AH Greenwood JS Dengler NG Programmed cell death remodels lace plant leaf shape during development Plant Cell 2004 16 60 73 10.1105/tpc.016188 14688291 PMC301395

[B8] Xu Y Hanson MR Programmed cell death during pollination-induced petal senescence in petunia Plant Physiol 2000 122 1323 1334 10.1104/pp.122.4.1323 10759529 PMC58968

[B9] Cacas JL Devil inside: does plant programmed cell death involve the endomembrane system? Plant Cell Environ 2010 33 1453 1473 20082668 10.1111/j.1365-3040.2010.02117.x

[B10] Jwa NS Park SG Park CH Kim SO Ahn IP Park SY Yoon CH Lee YH Cloning and expression of a rice cDNA encoding a Lls1 homologue of maize Plant Pathol J 2000 16 151 155

[B11] Chung E Oh SK Park JM Choi D Expression and promoter analyses of pepper CaCDPK4 (*Capsicum annuum *calcium dependent protein kinase 4) during plant defense response to incompatible pathogen Plant Pathol J 2007 23 76 89 10.5423/PPJ.2007.23.2.076

[B12] Zhou J Loh Y Bressan R Martin G The tomato gene Pti1 encodes a serine/threonine kinase that is phosphorylated by Pto and is involved in the hypersensitive response Cell 1995 83 925 935 10.1016/0092-8674(95)90208-2 8521516

[B13] Mittler R Rizhsky L Transgene-induced lesion mimic Plant Mol Biol 2000 44 335 344 10.1023/A:1026544625898 11199392

[B14] Vailleau F Daniel X Tronchet M Montillet JL Triantaphylidès C Roby D A R2R3-MYB gene, *AtMYB30*, acts as a positive regulator of the hypersensitive cell death program in plants in response to pathogen attack Proc Natl Acad Sci USA 2002 99 10179 10184 10.1073/pnas.152047199 12119395 PMC126644

[B15] Nadimpalli R Yalpani N Johal GS Simmons CR Prohibitins, stomatins, and plant disease response genes compose a protein superfamily that controls cell proliferation, ion channel regulation, and death J Biol Chem 2000 275 29579 29586 10.1074/jbc.M002339200 10862763

[B16] Rostoks N Schmierer D Kudrna D Kleinhofs A Barley putative hypersensitive induced reaction genes: genetic mapping, sequence analyses and differential expression in disease lesion mimic mutants Theor Appl Genet 2003 107 1094 1101 10.1007/s00122-003-1351-8 12928776

[B17] Xiao F Tang X Zhou JM Expression of 35S:: Pto globally activates defense-related genes in tomato plants Plant Physiol 2001 126 1637 1645 10.1104/pp.126.4.1637 11500562 PMC117163

[B18] Zhou L Cheung MY Zhang Q Lei CL Zhang SH Sun SSM Lam HM A novel simple extracellular leucine-rich repeat (eLRR) domain protein from rice (OsLRR1) enters the endosomal pathway and interacts with the hypersensitive-induced reaction protein 1 (OsHIR1) Plant Cell Environ 2009 32 1804 1820 10.1111/j.1365-3040.2009.02039.x 19712067

[B19] Yu XM Yu XD Qu ZP Huang XJ Guo J Han QM Zhao J Huang LL Kang ZS Cloning of a putative hypersensitive induced reaction gene from wheat infected by stripe rust fungus Gene 2008 407 193 198 10.1016/j.gene.2007.10.010 17980516

[B20] Jung HW Hwang BK The leucine-rich repeat (LRR) protein, CaLRR1, interacts with the hypersensitive induced reaction (HIR) protein, CaHIR1, and suppresses cell death induced by the CaHIR1 protein Mol Plant Pathol 2007 8 503 514 10.1111/j.1364-3703.2007.00410.x 20507517

[B21] Jaquinod M Villiers F Kieffer-Jaquinod S Hugouvieux V Bruley C Garin J Bourguignon J A proteomics dissection of *Arabidopsis thaliana *vacuoles isolated from cell culture Mol Cell Proteomics 2007 6 394 412 17151019 10.1074/mcp.M600250-MCP200 PMC2391258

[B22] Carter C Pan S Zouhar J Avila EL Girke T Raikhel NV The vegetative vacuole proteome of *Arabidopsis thaliana *reveals predicted and unexpected proteins Plant Cell 2004 16 3285 3303 10.1105/tpc.104.027078 15539469 PMC535874

[B23] De Castro E Sigrist CJA Gattiker A Bulliard V Langendijk-Genevaux PS Gasteiger E Bairoch A Hulo N ScanProsite: detection of PROSITE signature matches and ProRule-associated functional and structural residues in proteins Nucl Acids Res 2006 34 W362 W365 10.1093/nar/gkl124 16845026 PMC1538847

[B24] Ren J Wen L Gao X Jin C Xue Y Yao X CSS-Palm 2.0: an updated software for palmitoylation sites prediction Protein Eng Des Sel 2008 21 639 644 10.1093/protein/gzn039 18753194 PMC2569006

[B25] Thomma BPHJ Eggermont K Penninckx IAMA Mauch-Mani B Vogelsang R Cammue B Broekaert WF Separate jasmonate-dependent and salicylate-dependent defense-response pathways in Arabidopsis are essential for resistance to distinct microbial pathogens Proc Natl Acad Sci USA 1998 95 15107 15111 10.1073/pnas.95.25.15107 9844023 PMC24583

[B26] Browman DT Hoegg MB Robbins SM The SPFH domain-containing proteins: more than lipid raft markers Trends Cell Biol 2007 17 394 402 10.1016/j.tcb.2007.06.005 17766116

[B27] Lin SS Martin R Mongrand S Vandenabeele S Chen KC Jang IC Chua NH RING1 E3 ligase localizes to plasma membrane lipid rafts to trigger FB1-induced programmed cell death in Arabidopsis Plant J 2008 56 550 561 10.1111/j.1365-313X.2008.03625.x 18643987

[B28] Chen F Yuan Y Li Q He Z Proteomic analysis of rice plasma membrane reveals proteins involved in early defense response to bacterial blight Proteomics 2007 7 1529 1539 10.1002/pmic.200500765 17407182

[B29] Nohzadeh Malakshah S Habibi Rezaei M Heidari M Hosseini Salekdeh G Proteomics reveals new salt responsive proteins associated with rice plasma membrane Biosci Biotechnol Biochem 2007 71 2144 2154 10.1271/bbb.70027 17827676

[B30] Natera SHA Ford KL Cassin AM Patterson JH Newbigin EJ Bacic A Analysis of the *Oryza sativa *plasma membrane proteome using combined protein and peptide fractionation approaches in conjunction with mass spectrometry J Proteome Res 2008 7 1159 1187 10.1021/pr070255c 18260611

[B31] Marmagne A Ferro M Meinnel T Bruley C Kuhn L Garin J Barbier-Brygoo H Ephritikhine G A high content in lipid-modified peripheral proteins and integral receptor kinases features in the Arabidopsis plasma membrane proteome Mol Cell Proteomics 2007 6 1980 10.1074/mcp.M700099-MCP200 17644812

[B32] Hatsugai N Iwasaki S Tamura K Kondo M Fuji K Ogasawara K Nishimura M Hara-Nishimura I A novel membrane fusion-mediated plant immunity against bacterial pathogens Genes Dev 2009 23 2496 2506 10.1101/gad.1825209 19833761 PMC2779742

[B33] Ausubel FM Brent R Kingston RE Moore DD Seidman JG Smith JA Struhl K Phenol/SDS method for plant RNA preparation 1995 John Wiley & Sons Inc

[B34] Cheung MY Zeng NY Tong SW Li FWY Zhao KJ Zhang Q Sun SSM Lam HM Expression of a RING-HC protein from rice improves resistance to *Pseudomonas syringae *pv. *tomato *DC3000 in transgenic *Arabidopsis thaliana* J Exp Bot 2007 58 4147 4159 10.1093/jxb/erm272 18182423

[B35] Sambrook J Russell DW Molecular Cloning: a Laboratory Manual 2001 Cold Spring Harbor: Cold Spring Harbor Laboratory Press

[B36] Remans T Smeets K Opdenakker K Mathijsen D Vangronsveld J Cuypers A Normalisation of real-time RT-PCR gene expression measurements in *Arabidopsis thaliana *exposed to increased metal concentrations Planta 2008 227 1343 1349 10.1007/s00425-008-0706-4 18273637

[B37] Jain M Nijhawan A Tyagi AK Khurana JP Validation of housekeeping genes as internal control for studying gene expression in rice by quantitative real-time PCR Biochem Biophys Res Commun 2006 345 646 651 10.1016/j.bbrc.2006.04.140 16690022

[B38] Livak KJ Schmittgen TD Analysis of relative gene expression data using real-time quantitative PCR and the 2(-Delta Delta C(T)) method Methods 2001 25 402 408 10.1006/meth.2001.1262 11846609

[B39] Finckh U Lingenfelter PA Myerson D Producing single-stranded DNA probes with the Taq DNA polymerase: a high yield protocol BioTechniques 1991 10 35 36 38-39 2003918

[B40] Cheung MY Zeng NY Tong SW Li FWY Xue Y Zhao KJ Wang C Zhang Q Fu Y Sun Z Constitutive expression of a rice GTPase-activating protein induces defense responses New Phytol 2008 179 530 545 10.1111/j.1469-8137.2008.02473.x 19086295

[B41] Zhang Q Shi AN Yang WC Wang CL Breeding of three near-isogenic Japonica rice lines with different major genes for resistance to bacterial blight Acta Agronom Sin 1996 22 135 141

[B42] Katagiri F Thilmony R He SY Somerville CR, Meyerowitz EM The *Arabidopsis thaliana-Pseudomonas syringae *interaction The Arabidopsis Book 2002 39 Rockville, MD: American Society of Plant Biologists 10.1199/tab.0039 PMC3243347 22303207

[B43] Yu HX Liu QQ Wang L Zhao ZP Xu L Huang BL Gong ZY Tang SZ Gu NH Breeding of selectable marker-free transgenic rice lines containing AP1 gene with enhanced disease resistance Sci Agric Sin 2006 5 805 811

[B44] Rooke L Byrne D Salgueiro S Marker gene expression driven by the maize ubiquitin promoter in transgenic wheat Ann Appl Biol 2000 136 167 172 10.1111/j.1744-7348.2000.tb00022.x

[B45] Hiei Y Ohta S Komari T Kumashiro T Efficient transformation of rice (*Oryza sativa L*.) mediated by Agrobacterium and sequence analysis of the boundaries of the T-DNA Plant J 1994 6 271 282 10.1046/j.1365-313X.1994.6020271.x 7920717

[B46] Zhu L Fu YP Liu WZ Hu GC Si HM Tang KX Sun ZX Rapidly obtaining the marker-free transgenic rice with three target genes by co-transformation an anther culture Chin J Rice Sci 2007 14 239 247 10.1016/S1672-6308(08)60001-3

[B47] Brears T Liu C Knight TJ Coruzzi GM Ectopic overexpression of asparagine synthetase in transgenic tobacco Plant Physiol 1993 103 1285 1290 12232020 10.1104/pp.103.4.1285 PMC159117

[B48] Bent AF Arabidopsis *in planta *transformation. Uses, mechanisms, and prospects for transformation of other species Plant Physiol 2000 124 1540 1547 10.1104/pp.124.4.1540 11115872 PMC1539310

[B49] Lam SK Siu CL Hillmer S Jang S An G Robinson DG Jiang L Rice SCAMP1 defines clathrin-coated, trans-Golgi-located tubular-vesicular structures as an early endosome in tobacco BY-2 cells Plant Cell 2007 19 296 319 10.1105/tpc.106.045708 17209124 PMC1820953

[B50] Koch E Slusarenko A Arabidopsis is susceptible to infection by a downy mildew fungus Plant Cell 1990 2 437 445 10.1105/tpc.2.5.437 2152169 PMC159900

[B51] Claros MG Heijne G TopPred II: an improved software for membrane protein structure predictions Comp Appl Biosci 1994 10 685 686 7704669 10.1093/bioinformatics/10.6.685

